# Determining the Ensemble *N*‑Representability of Reduced Density Matrices

**DOI:** 10.1021/acs.jctc.5c01788

**Published:** 2025-12-26

**Authors:** Ofelia B. Oña, Gustavo E. Massaccesi, Pablo Capuzzi, Luis Lain, Alicia Torre, Juan E. Peralta, Diego R. Alcoba, Gustavo E. Scuseria

**Affiliations:** † Instituto de Investigaciones Fisicoquímicas Teóricas Y Aplicadas, 62873 Universidad Nacional de La Plata, Consejo Nacional de Investigaciones Científicas Y Técnicas, Diag. 113 Y 64 (S/N), Sucursal 4, CC 16, La Plata 1900, Argentina; ‡ Departamento de Ciencias Exactas, Ciclo Básico Común, Universidad de Buenos Aires, Ciudad Universitaria, Buenos Aires 1428, Argentina; § Instituto de Investigaciones Matemáticas “Luis A. Santaló” (IMAS), Consejo Nacional de Investigaciones Científicas Y Técnicas, Universidad de Buenos Aires, Ciudad Universitaria, Buenos Aires 1428, Argentina; ∥ 28196 Universidad de Buenos Aires, Facultad de Ciencias Exactas Y Naturales, Departamento de Física, Ciudad Universitaria, Buenos Aires 1428, Argentina; ⊥ CONICET - Universidad de Buenos Aires, Instituto de Física de Buenos Aires (IFIBA), Ciudad Universitaria, Buenos Aires 1428, Argentina; # Department of Physical Chemistry, Faculty of Science and Technology, 16402 University of the Basque Country, PO Box 644, Bilbao E-48080, Spain; ∇ Department of Physics, 5649 Central Michigan University, Mount Pleasant, Michigan 48859, United States; ○ Department of Chemistry, 3990 Rice University, Houston, Texas 77005-1892, United States; ◆ Department of Physics and Astronomy, Rice University, Houston, Texas 77005-1892, United States

## Abstract

The *N*-representability problem for reduced density matrices remains a fundamental challenge in electronic structure theory. Following our previous work that employs a unitary-evolution algorithm based on an adaptive derivative-assembled pseudo-Trotter variational quantum algorithm to probe *pure-state N*-representability of reduced density matrices [J. Chem. Theory Comput. 2024, 20, 9968], in this work we propose a practical framework for determining the *ensemble N*-representability of a *p*-body matrix. This is accomplished using a purification strategy that embeds an ensemble state into a pure state defined on an extended Hilbert space, such that the reduced density matrices of the purified state reproduce those of the original ensemble. By iteratively applying variational unitaries to an initial purified state, the proposed algorithm minimizes the Hilbert-Schmidt distance between its *p*-body reduced density matrix and a specified target *p*-body matrix, which serves as a measure of the *N*-representability of the target. This methodology facilitates both error correction of defective ensemble reduced density matrices and quantum-state reconstruction on a quantum computer, offering a route for density-matrix refinement. We validate the algorithm with numerical simulations on systems of two, three, and four electrons in both simple models as well as molecular systems at finite temperature, demonstrating its robustness.

## Graphical abstract

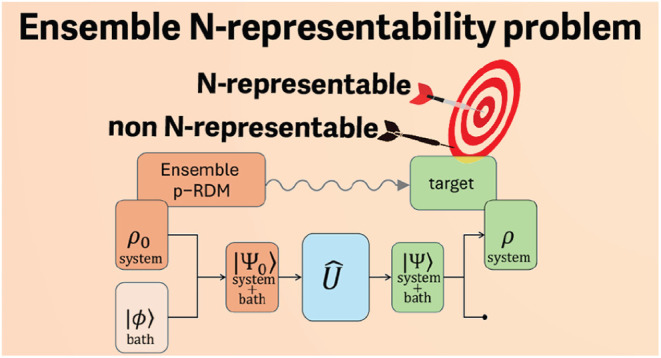

## Introduction

The significance of the *N*-representability problem in electronic structure theory is both foundational and far-reaching.^1^ At the heart of this challenge lies the quest to determine the necessary and sufficient conditions that a *p*-body reduced density matrix (*p*-RDM) must satisfy to be derivable from a *N*-electron wave function or mixed (ensemble) state.^2–9^ The special case of the 2-RDM is essential for the development of quantum chemistry computational methods.^2,3,10^ Equally important, though often less emphasized, is the role of the 1-RDM in practical applications, such as in Hartree–Fock and density (matrix) functional theory, where the 1-RDM serves as a cornerstone for calculating observable properties.^11–13^ Despite its theoretical interest, the *N*-representability problem presents formidable computational challenges. It belongs to the quantum Merlin-Arthur-complete class of problems: a quantum analog of the well-known nondeterministic polynomial-time complete class in classical complexity theory.^14^ This classification implies that, in general, verifying whether a given *p*-RDM corresponds to a physical *N*-electron state is computationally intractable, especially as system size increases. Recent developments have led to sets of approximate *N*-representability conditions that, while not exhaustive, are sufficiently robust to enable meaningful calculations for strongly correlated electron systems, particularly in small to medium-sized molecules.^10,15–20^ Moreover, Coleman^4^ and Klyachko^21,22^ derived the necessary and sufficient *N*-representability conditions that must be fulfilled by the 1-RDM to be derivable from an ensemble or pure *N*-Fermion quantum state, respectively. These conditions, which constrain the eigenvalues of the 1-RDM, offer a mathematically rigorous framework for identifying physically valid Fermionic occupation numbers.^2,22–31^

In recent work, we introduced an algorithm^32^ designed to numerically determine whether a given *p*-body reduced density matrix (*p*-RDM) is pure *N*-representable, or in other words, whether it can be obtained by tracing out (*N*–*p*) degrees of freedom from a pure *N*-body quantum state (i.e., a single wave function or pure-state density matrix). The proposed algorithm operates by guiding the unitary evolution of an ansatz wave function through its corresponding *p*-RDM toward a target matrix (alleged *p*-RDM), with the goal of minimizing their Hilbert-Schmidt distance (a measure of how close two matrices are in Hilbert space). The evolution (driven by stochastic sampling in that work) is inspired by the adaptive derivative-assembled pseudo-Trotter (ADAPT) framework, originally developed for the variational quantum eigensolver (VQE) problem,^33^ and other approaches related to it, including the contracted quantum eigensolver.^34–41^ As such, the algorithm is suited for implementation on quantum devices.^33,42,43^ We refer to this approach as pure ADAPT-VQA (Variational Quantum Algorithm), and its utility extends beyond mere classification. Not only can it determine whether a given matrix is a valid pure *p*-RDM, but it can also correct matrices that fail to meet the *N*-representability criteria. This dual capability makes the pure ADAPT-VQA a powerful tool for quantum simulations, offering a pathway to enforce physical constraints in quantum algorithms and potentially improve the accuracy of quantum chemical calculations.

In this work, we address a critical extension of the *N*-representability problem, namely, its formulation for ensemble *p*-RDMs.^2,3^ While part of the existing literature focuses on pure-state representability,^2,22–30,44^ ensemble states are equally important, especially in contexts involving thermal mixtures, open quantum systems, and statistical ensembles.^2,3^ To address this challenge, we employ a purification-based method in which the ensemble state is represented as a pure state within an expanded Hilbert space,^45,46^ which has been recently applied to finite temperature electronic systems through the thermofield theory.^47–49^ This method ensures that the reduced density matrices of both the ensemble and its purified counterpart are identical, allowing us to leverage techniques developed for pure states. Specifically, we apply the pure ADAPT-VQA algorithm introduced in our previous work,^32^ which enables the unitary evolution of the purified state to minimize the Hilbert-Schmidt distance between its *p*-RDM and a given target matrix. Building on this foundation, we introduce a new algorithm, the ensemble ADAPT-VQA, which extends the capabilities of its pure-state predecessor. This hybrid framework allows us not only to determine whether a *p*-body matrix is *N*-representable, but also to discern the nature of its origin: whether it arises from a pure quantum state or from an ensemble mixture of pure states. Such a distinction is crucial for interpreting quantum simulations and for enforcing physical constraints in variational algorithms. To validate the performance and versatility of ensemble ADAPT-VQA, we conduct numerical experiments on both model and molecular systems. Specifically, we test the algorithm on the 1-RDM and 2-RDM of four-electron systems at zero temperature, as well as on the H_2_ and H_3_ molecules at finite temperature. These examples demonstrate the ability of the algorithm to handle pure and thermal-state scenarios, highlighting its potential for broader applications in quantum chemistry and quantum information.

## Theoretical Considerations

Let us consider a quantum mixed (ensemble) state, ρ, defined on a finite-dimensional Hilbert space $H_{s}$ , which can be expressed as

1 $$\rho =\underset{i}{\sum }p_{i}\left|\phi_{i}\right\rangle \left\langle \phi_{i}\right|$$

where $\left|\phi_{i}\right\rangle \in H_{s}$ are pure-state wave functions, and *p*_*i*_ probabilities satisfying *p*_*i*_ ≥ 0 with ∑_*i*_ *p*_*i*_ = 1. According to the Schrödinger-Hughston-Jozsa-Wootters theorem,^50^ any such mixed state ρ admits a purification: there exists an auxiliary finite-dimensional Hilbert space $H_{b}$ and a pure-state wave function $\left|\Psi_{sb}\right\rangle \in H_{sb}$ (with $H_{sb}=H_{s}⊗H_{b}$ an extended Hilbert space), referred to as a purification of ρ, such that^45^

2 $$\rho ={\mathrm{Tr}}_{b}\left(\left|\Psi_{sb}\right\rangle \langle \Psi_{sb}|\right)$$

where Tr_*b*_ implies the complete trace over the degrees of freedom on the auxiliary Hilbert space $H_{b}$ . Moreover, every purification of ρ can be written in the form

3 $$\left|\Psi_{sb}\right\rangle =\underset{i}{\sum }\sqrt{p_{i}}\left|\phi_{i}\right\rangle ⊗\left|b_{i}\right\rangle$$

where {|*b*_*i*_⟩}_*i*_ is an orthonormal basis of $H_{b}$ . Thus, one can use this strategy to embed an ensemble state into a pure state defined in an extended Hilbert space, such that the *p*-RDMs of the purified ensemble are reproduced by those of the pure state.

Equation 3 provides a practical approach to numerically determine the ensemble *N*-representability of a *p*-RDM by unitarily evolving a pure state. This can be accomplished using an algorithm that has been recently introduced by us to deal with the pure *N*-representability problem.^32^ Here we summarize the newly proposed algorithm employed for the ensemble case. The underlying idea is to find a sequence of unitary transformations that evolve an initial 2*N*-particle wave function |Ψ_0_⟩ built according to eq 3, e.g.,

4 $$\left|\Psi_{0}\right\rangle =\left|\phi_{0}\right\rangle ⊗\left|\phi_{0}\right\rangle$$

with |ϕ_0_⟩ holding *N* particles. The evolution is such that the Hilbert-Schmidt distance D between the *p*-RDM corresponding to the evolved state |Ψ_*k*_⟩,

5 ρkp=p!(Np)TrN−p(Trb(|Ψk⟩⟨Ψk|))

and a given (possibly) ensemble *N*-representable target *p*-RDM, ^*p*^ρ_target_, is minimized. The (squared) distance is calculated according to the Hilbert–Schmidt norm,

6 Dk=∥ρkp−ρtargetp∥22

At convergence, the ADAPT-VQA yields a minimum feasible distance for the problem at hand, $D_{\min}$ , and the state |Ψ_min_⟩. Thus, if the algorithm evolves the distance $D_{k}$ to numerical zero, then ^*p*^ρ_target_ is ensemble *N*-representable. In general, $D_{\min}$ can be considered as a measure of the degree of ensemble *N*-representability of ^*p*^ρ_target_, and the evolved ^*p*^ρ is an ensemble *N*-representable (physical) reduced state that is closest to ^*p*^ρ_target_. Hence, the algorithm allows to correct the possible error associated with the lack of ensemble *N*-representability of ^*p*^ρ_target_.

The minimization algorithm employed in this work is schematized in Algorithm 1. The procedure follows the schemes previously introduced by us to determine the *N*-representability of pure RDMs and transition RDMs.^32,51^ In the present work, we adopt the same gradient-based strategy utilized for transition RDMs, in combination with an antihermitian excitation-deexcitation operator pool {*Ô*} suitable for Fermions,^52–55^

7 $${Ô}_{ik}={â}_{i}^{†}{â}_{k}-{â}_{k}^{†}{â}_{i}$$

and

8 $${Ô}_{ijkl}={â}_{i}^{†}{â}_{j}^{†}{â}_{k}{â}_{l}-{â}_{k}^{†}{â}_{l}^{†}{â}_{i}{â}_{j}$$

for one- and two-particle excitations, respectively. In eqs 7 and 8, *â* ^†^ and *â* are the usual Fermionic creation and annihilation operators acting on an orthonormal finite single-particle basis associated with the extended Hilbert space $H_{sb}$ . Furthermore, since we opt to work on a canonical ensemble framework, the operator pool is restricted in such a way that no electronic transitions changing the number of particles in the system of interest nor in the auxiliary system are allowed. In practice, the operators in eqs 7 and 8 are represented as a combination of Pauli operators via the Jordan-Wigner transformation,^56^ ensuring that the proposed algorithm is suitable for simulations on quantum computers.^57^ 
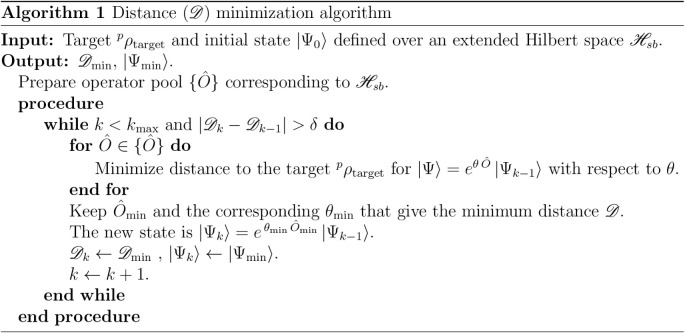


## Computational Details

The implementation of the algorithm described in the previous section utilizes an in-house code written in Python that uses OpenFermion^58^ for the Jordan-Wigner mapping and the OpenFermion module of PySCF^59,60^ for integral computation and manipulation. Computations reported in this work are carried out on a simulated noiseless quantum device. The original ensemble *N*-electron problem is embedded into a extended pure 2*N*-electron problem (eq 3). The former uses *K* spatial orbitals as single-particle basis, while the latter uses 2*K* orbitals. For the 4-electron model systems at zero temperature, we use *K* = 3 and *K* = 4 single-particle spatial orbitals, referred to as (4*e*, 3*o*) and (4*e*, 4*o*) hereafter, while for the H_2_ and H_3_ molecular systems at finite temperature we use STO-3G basis set (*K* = 2 and *K* = 3, respectively). This choice allows us to deal with 1- and 2-RDMs, which are commonly the most relevant for electronic structure problems, while keeping the computations tractable. This leads to a pool of 378 and 1196 operators for the (4*e*, 3*o*), (4*e*, 4*o*), respectively, while for the H_2_ and H_3_ molecular cases the number of operators in the pool is 72, and 378, respectively. The convergence threshold, δ, in the iterative process (Algorithm 1) is varied within the range [5 × 10^–9^, 3 × 10^–5^]. A smaller δ is used for stricter convergence requirements, such as for *N*-representable one-body targets, while δ is increased for two-body targets and in cases of a stronger *N*-representability violation. Details of the number of iterations to reach convergence are available in the (Supporting Information SI).

## Results and Discussion

### Model Systems at Zero Temperature

Given a *p*-RDM, one can determine if it is pure *N*-representable utilizing the previously proposed pure ADAPT-VQA. If the *p*-RDM results non pure *N*-representable, one can utilize the ensemble ADAPT-VQA proposed in this work to determine its ensemble *N*-representability. Thus, the two algorithms serve to classify a *p*-RDM as pure or ensemble *N*-representable. We first test this idea to numerically determine if the 1- and 2-RDMs of two analytical model systems are pure or ensemble *N*-representable. To assess the validity of our numerical results, we make use of Klyachko’s *N*-representability inequalities (also known as generalized Pauli conditions), which provide a set of necessary and sufficient analytical constraints that pure 1-RDMs must satisfy.^21,22^ For the (4*e*, 3*o*) and (4*e*, 4*o*) model systems, we construct target *p*-RDMs as

9 ρtargetp=p!(Np)TrN−p(ρ)

where the state ρ is a convex linear combination of two pure states, ρ_1_ and ρ_2_ (shown in Table 1),

10 $$\rho =w\rho_{1}+\left(1-w\right)\rho_{2},0\leq w\leq 1$$

The parameter *w* in eq 10 is set to a value of 0.0 or 0.5 for ρ representing either a pure, or mixed (ensemble) state, respectively. This choice is such that Klyachko’s conditions in the (4*e*, 3*o*) model system are satisfied for both *w* = 0.0 and *w* = 0.5, whereas in the (4*e*, 4*o*) case, these conditions are satisfied only for *w* = 0.0 (see Table S1 in the SI for the complete list of inequalities). Table 2 shows our results for the 1-RDM of the (4*e*, 3*o*) and (4*e*, 4*o*) model systems for *w* = 0.0 and *w* = 0.5. In all cases, the initial state is taken as ρ_1_ for the pure case, or its purification for the ensemble case. For the (4*e*, 3*o*) model system, the pure and ensemble ADAPT-VQA yield converged $D_{\min}$ that are numerically zero for both *w* values, indicating that there exists pure states that are compatible with the target 1-RDMs. This is consistent with the satisfaction of all Klyachko’s conditions, as shown in Table 2. For the (4*e*, 4*o*) model system, on the other hand, both the pure and ensemble ADAPT-VQAs yield converged $D_{\min}\approx 0$ for *w* = 0.0, while for *w* = 0.5 (for which Klyachko’s conditions are not fulfilled), $D_{\min}$ approaches zero only for the ensemble algorithm. This highlights the ability of the algorithms to discern the pure or ensemble *N*-representability nature of 1-RDMs. Table 2 also reports the number of Klyachko’s inequalities that are satisfied for each case. We note that all targets are identified as ensemble *N*-representable by the ensemble ADAPT-VQA, in agreement with the fulfillment of Coleman’s ensemble *N*-representability conditions (1-RDM eigenvalues between 0 and 1, and tracing to the number of electrons).^4^

**Table 1:** States Used to Construct the Target RDMs for the (4*e*, 3*o*) and (4*e*, 4*o*) Model Systems in eq 2

	State	
Model system	ρ_1_	ρ_2_
(4*e*, 3*o*)	|1^α^ 1^β^ 2^α^ 2^β^⟩	|1^α^ 1^β^ 3^α^ 3^β^⟩
(4*e*, 4*o*)	|1^α^ 1^β^ 2^α^ 2^β^⟩	|1^α^ 1^β^ 3^α^ 2^β^⟩

**Table 2:** Converged $D_{\min}$ from the Pure and Ensemble ADAPT-VQA for 4-Electron Model Systems for 1-RDMs, Along with the 1-RDMs Eigenvalues, and the Number of Klyachko’s Pure *N*-Representability Inequalities That is Fulfilled in Each Case, *N*_ineq_ (Out of a Total of 15 for This Model)

	Model system			
	(4*e*, 3*o*)		(4*e*, 4*o*)	
1-RDM eigenvalues	*w* = 0.0	*w* = 0.5	*w* = 0.0	*w* = 0.5
λ_1_	1.000	1.000	1.000	1.000
λ_2_	1.000	1.000	1.000	1.000
λ_3_	1.000	0.500	1.000	1.000
λ_4_	1.000	0.500	1.000	0.500
λ_5_	0.000	0.500	0.000	0.500
λ_6_	0.000	0.500	0.000	0.000
λ_7_			0.000	0.000
λ_8_			0.000	0.000
*N*_ineq_	15	15	15	7
ADAPT-VQA	$D_{\min}$			
Pure	0.0	4.89 × 10^–9^	0.0	1.25 × 10^–1^
Ensemble	0.0	4.89 × 10^–9^	0.0	2.45 × 10^–9^

Next, we extend the analysis to 2-RDMs. While a set of necessary pure *N*-representability conditions has been reported for 2-RDMs,^44^ the complete set of necessary and sufficient pure *N*-representability conditions remains unknown. Our ADAPT-VQA can be used to numerically decide the pure or ensemble *N*-representability of 2-RDMs. To highlight this, we analyze the *N*-representability of 2-RDMs for the (4*e*, 3*o*) and (4*e*, 4*o*) model systems. Our results are summarized in Table 3. For these model systems, the algorithm identifies all the 2-RDMs as ensemble *N*-representable, but only the cases corresponding to *w* = 0.0 are identified as pure *N*-representable, as expected. This brings an interesting question: Can the method be used to detect if an ensemble *N*-representable *p*-RDM would contract to a pure *N*-representable *q*-RDM (*q* < *p*)? To answer this question, we build target RDMs for a (4*e*, 4*o*) model system from an ensemble state as linear combination of two pure states (eq 10 with *w* = 0.5), which are in turn constructed as shown in Table 4. These states are chosen so that they differ in one, two, or three spin–orbitals. A summary of the results is shown in Table 5. For the case of triple substitution, the 1-RDM and the 2-RDM can be also obtained from a wave function (a linear combination of those reported in Table 4), and thus they are both pure and ensemble *N*-representable. This indicates that the *N*-electron state that generated these RDMs cannot be uniquely determined solely from them. Thus, $D_{\min}$ is expected to approach zero as the pure and ensemble algorithms evolve. The numerical results shown in Table 5 confirm that the ADAPT-VQA yields the expected outcomes. For the doubly substituted case, only the pure algorithm yields $D_{\min}\neq 0$ for the 2-RDM, showing that indeed this matrix corresponds to an ensemble state, while its contraction leads to a 1-RDM that can be identified as pure *N*-representable. For completeness, Table 5 shows the results for the single substitution case, indicating that neither the target 1-RDM nor the target 2-RDM can be obtained from a pure state. It is important to emphasize that, although the 1- and 2-RDMs are constructed from an ensemble state, the pure algorithm is able to find a compatible wave function that yields the same RDMs (provided that $D_{\min}\to 0$ ), highlighting its potential use in quantum tomography.^61^

**Table 3:** Converged $D_{\min}$ from the Pure and Ensemble ADAPT-VQA for the (4*e*, 3*o*) and (4*e*, 4*o*) Model Systems for 2-RDMs

	$$D_{\min}$$			
	(4*e*, 3*o*)		(4*e*, 4*o*)	
Algorithm	*w* = 0.0	*w* = 0.5	*w* = 0.0	*w* = 0.5
Pure	0.0	2.00	0.0	4.25
Ensemble	1.07 × 10^–14^	3.11 × 10^–12^	1.07 × 10^–14^	1.07 × 10^–14^

**Table 4:** Singly- (1), Doubly- (2), and Triply- (3) Spin-Orbital-Substituted States Used to Construct the Target RDMs for the (4*e*, 4*o*) Model System

Substitution	ρ_1_	ρ_2_
1	|1^α^ 1^β^ 2^α^ 2^β^⟩	|1^α^ 1^β^ 3^α^ 2^β^⟩
2	|1^α^ 1^β^ 2^α^ 2^β^⟩	|1^α^ 1^β^ 3^α^ 3^β^⟩
3	|1^α^ 1^β^ 2^α^ 2^β^⟩	|1^α^ 3^β^ 4^α^ 4^β^⟩

**Table 5:** Converged $D_{\min}$ from the Pure and Ensemble ADAPT-VQA for the (4*e*, 4*o*) Model System for 1- and 2-RDMs with *w* = 0.5. The Targets are the (Ensemble) Reduced Density Matrices Corresponding to the States Constructed from Multiple Substitutions as Shown in Table 4

	$$D_{\min}$$			
	1-RDM		2-RDM	
	Algorithm			
Substitution	Pure	Ensemble	Pure	Ensemble
1	1.25 × 10^–1^	2.45 × 10^–9^	4.25	1.07 × 10^–14^
2	4.89 × 10^–9^	4.89 × 10^–9^	2.00	9.66 × 10^–9^
3	2.45 × 10^–9^	2.45 × 10^–9^	1.42 × 10^–14^	2.77 × 10^–8^

So far we have tested our ADAPT-VQA with *N*-representable RDMs as targets. To test the ensemble algorithm with cases where the *N*-representability is violated, we have incorporated numerical noise to RDMs, ^*p*^ρ, using

11 ρtargetp(ε)=ρp+εR

where *R* is a matrix of random numbers taken from a uniform probability distribution in [−1, 1] and *ε* is in the interval [0, 0.1]. In eq 11, ^*p*^ρ is constructed according to eq 10 with *w* = 0.5 and ρ_1_ and ρ_2_ given in Table 1. The values of *ε* are chosen so that the resulting noise produces noticeably *N*-representability defects on ^*p*^ρ_target_(*ε*). Thus, the algorithm should evolve the initial RDM toward the target up to a certain limit, depending on the strength of the noise. Larger noise strengths should lead to larger $D_{\min}$ , while a noiseless target should converge $D_{\min}\to 0$ as previously shown. Table 6 shows the converged $D_{\min}$ for ^1^ρ_target_(*ε*) and ^2^ρ_target_(*ε*) corresponding to the (4*e*, 4*o*) model system for different noise strengths. The converged $D_{\min}$ in Table 6 can then be considered as a numerical measure of the violation of the ensemble *N*-representability. These results are in line with the findings in ref 32 for the pure ADAPT-VQA when considering systematic violations of *N*-representability conditions. This emphasizes applications for the ADAPT-VQA: It can be used not only to determine the ensemble *N*-representability of an alleged *p*-RDM, but also to construct an ensemble *p*-RDM (the evolved RDM) that is closest to the target ^*p*^ ρ_target_.

**Table 6:** Converged $D_{\min}$ from the Ensemble ADAPT-VQA for the 1- and 2-RDMs in the (4*e*, 4*o*) Model System^a^

	$$D_{\min}$$	
*ε*	1-RDM	2-RDM
0.0	2.45 × 10^–9^	1.07 × 10^–14^
10^–2^	2.02 × 10^–3^	1.38 × 10^–1^
10^–1^	2.19 × 10^–1^	1.33 × 10^1^

a The targets are constructed by adding random noise of strength *ε* to the reduced density matrices (see text for details).

### Molecular Systems at Finite Temperature

We next assess the usability of the ensemble ADAPT-VQA for H_2_ and linear H_3_ molecular systems at a finite temperature. In this case, the *p*-RDMs are constructed from their canonical ensemble thermal states at temperature *T*,

12 $$\rho =Z^{-1}\underset{i}{\sum }e^{-E_{i}/k_{B}T}\rho_{i}$$

where *k*_B_ is the Boltzmann constant, and the canonical partition function *Z* is given by

13 $$Z=\underset{i}{\sum }e^{-E_{i}/k_{B}T}$$

In eq 12, *E*_*i*_ and ρ_*i*_ are the eigenenergies and eigenstates of the electronic Hamiltonian *H*, which can be written in the second quantization formalism as^62^

14 $$Ĥ=\underset{ij}{\sum }\left\langle i\right|h\left|j\right\rangle {â}_{i}^{†}{â}_{j}+\frac{1}{4}\underset{ijkl}{\sum }\left\langle ij\right|v\left|kl\right\rangle {â}_{i}^{†}{â}_{j}^{†}{â}_{l}{â}_{k}$$

where ⟨*i*|*h*|*j*⟩ and ⟨*ij*|*v*|*kl*⟩ are the standard one- and two-electron antisymmetrized integrals, respectively.

We consider both molecular systems at two nuclear configurations: close to equilibrium (*R*_HH_ = 0.75 Å) and stretched (*R*_HH_ = 1.5 Å). In all cases *k*_B_ *T* is the energy gap between the ground and first excited states. With this choice, the stretched configuration presents a thermal distribution with larger weights for the lower energy states than that of the equilibrium configuration. Using the ensemble RDMs corresponding to these thermally weighted states, we construct target matrices by adding random noise of strength *ε* as described above (see eq 11). We observe that for H_2_, the 2-RDM is not a reduced state of the system. In all cases, the initial state has been constructed considering eq 4, with |ϕ_0_⟩ the Hartree–Fock ground state. In Table 7 we show the converged $D_{\min}$ for three values of *ε*. The results should serve as an indication of what to expect for the ensemble ADAPT-VQA for physical systems when the target matrices deviate from ideal ensemble *N*-representability. For example, when this deviation increases, as measured by the parameter *ε*, $D_{\min}$ also increases to values of ∼10^–2^ and ∼10^0^ for the 1- and 2-RDM for *ε* = 0.1, respectively. We note that this observation holds for both nuclear configurations, indicating that the converged $D_{\min}$ is more sensitive to the added noise than to the correlated characteristic of the underlying noiseless reduced state.

**Table 7:** Converged $D_{\min}$ from the Ensemble ADAPT-VQA for the Equilibrium and Stretched H_2_ and Linear H_3_ molecules^a^

	$$D_{\min}$$			
	*H*_2_		*H*_3_	
*ε*	1-RDM	2-RDM	1-RDM	2-RDM
*R*_HH_ = 0.75 Å				
0.0	0.0	4.95 × 10^–9^	4.45 × 10^–9^	1.98 × 10^–5^
10^–2^	3.11 × 10^–4^	8.12 × 10^–3^	1.08 × 10^–3^	4.24 × 10^–2^
10^–1^	3.55 × 10^–2^	7.71 × 10^–1^	9.27 × 10^–2^	4.08
*R*_HH_ = 1.5 Å				
0.0	0.0	1.76 × 10^–7^	4.11 × 10^–10^	4.73 × 10^–5^
10^–2^	4.08 × 10^–4^	7.67 × 10^–3^	9.40 × 10^–4^	4.44 × 10^–2^
10^–1^	4.95 × 10^–2^	7.98 × 10^–1^	6.47 × 10^–2^	4.20

a The targets are constructed by adding random noise of strength *ε* to the RDMs corresponding to canonical ensemble thermal states (see text for details).

## Summary

We have proposed and assessed a practical framework for determining the ensemble *N*-representability of an (alleged) *p*-body RDM. To this end, we employ a purification scheme that embeds an ensemble state into a pure state defined on an extended Hilbert space, in a way that the reduced density matrices of the purified state reproduce those of the original ensemble. We then utilize a recently reported unitary-evolution algorithm for pure RDMs to iteratively apply variational unitaries to an initial purified state. The algorithm effectively minimizes the distance between the corresponding *p*-body reduced density matrix and a specified target *p*-body matrix, alleged to be a proper ensemble *p*-RDM. This methodology can be used for both, correcting the lack of ensemble *N*-representability of target matrices and reconstructing a quantum-state from the corrected targets, offering a route for *p*-RDM refinement. Moreover, the proposed algorithm is compatible with quantum computers.

We have validated the algorithm with numerical simulations on systems of two, three, and four electrons in both, model problems and molecular examples. For model systems of four electrons in three and four orbitals, we have assessed the effectiveness of the algorithm to decide on the pure or ensemble nature of 1-RDMs, and compared against Klyachko’s pure *N*-representability conditions. For 2-RDMs, where the sufficient pure *N*-representability conditions are unknown, our algorithm offers a practical route for dealing with this problem. We have also considered the capability of the ADAPT-VQA to deal with cases where the target matrices may not be *N*-representable. To this end, we have generated target matrices by artificially breaking the *N*-representability of 1- and 2-RDMs in a model system, and in molecular H_2_ and H_3_ at finite temperature. Overall, the numerical results show that the proposed algorithm is effectively able to detect the type of *N*-representability (pure or ensemble) of a target matrix, or the lack of it, as well as provide a closest RDM to a given non-*N*-representable target matrix and the corresponding (pure or purified) state.

## Supplementary Material



## Notes

Klyachko’s pure *N*-representability inequalities for the (4*e*, 3*o*) and (4*e*, 4*o*) model systems, and the number of iterations required to achieve convergence for the various systems in this work (PDF)
