# Supplementary material for: Determining the Ensemble N‑Representability of Reduced Density Matrices
Source: J Chem Theory Comput. 2025 Dec 26;22(1):399–406. doi: 10.1021/acs.jctc.5c01788 (PMC12805516; doi:10.1021/acs.jctc.5c01788)
Supplement: Supplementary file 1 [file ct5c01788_si_001.pdf]

Supporting Information: Determining the ensemble N-representability of Reduced Density Matrices

Ofelia B. Oña,[†] Gustavo E. Massaccesi,[‡,¶] Pablo Capuzzi,[§,‖] Luis Lain,[⊥] Alicia Torre,[⊥] Juan E. Peralta,[*,#] Diego R. Alcoba,[*,§,‖] and Gustavo E. Scuseria[*,@,△]

†*Instituto de Investigaciones Fisicoquímicas Teóricas y Aplicadas, Universidad Nacional de La Plata, Consejo Nacional de Investigaciones Científicas y Técnicas. Diag. 113 y 64 (S/N), Sucursal 4, CC 16, 1900 La Plata, Argentina*

‡*Departamento de Ciencias Exactas, Ciclo Básico Común, Universidad de Buenos Aires, Ciudad Universitaria, 1428 Buenos Aires, Argentina*

¶*Instituto de Investigaciones Matemáticas "Luis A. Santaló" (IMAS), Consejo Nacional de Investigaciones Científicas y Técnicas, Universidad de Buenos Aires. Ciudad Universitaria, 1428 Buenos Aires, Argentina*

§*Universidad de Buenos Aires, Facultad de Ciencias Exactas y Naturales, Departamento de Física. Ciudad Universitaria, 1428 Buenos Aires, Argentina*

‖*CONICET - Universidad de Buenos Aires, Instituto de Física de Buenos Aires (IFIBA). Ciudad Universitaria, 1428 Buenos Aires, Argentina*

⊥*Department of Physical Chemistry, Faculty of Science and Technology, University of the Basque Country. PO Box 644, E-48080 Bilbao, Spain*

#*Department of Physics, Central Michigan University, Mount Pleasant, MI, 48859, USA*

@*Department of Chemistry, Rice University, Houston, TX 77005-1892*

△*Department of Physics and Astronomy, Rice University, Houston, TX 77005-1892*

E-mail: juan.peralta@cmich.edu; dalcoba@df.uba.ar; guscus@rice.edu

Supporting Information Available

Table S1: The complete list of Klyachko's pure N-representability inequalities, along with their fulfillment (✓) or violation (×), is shown for the 1-RDMs obtained from the pure and ensemble ADAPT-VQA calculations. The λ_i correspond to the eigenvalues of the 1-RDMs. (see Table 2 in the main manuscript)

Klyachko's inequality	Model system			
	$(4e, 3o)$		$(4e, 4o)$	
	$w = 0.0$	$w = 0.5$	$w = 0.0$	$w = 0.5$
$\lambda_1 \leq 1$	✓	✓	✓	✓
$\lambda_5 - \lambda_6 - \lambda_7 - \lambda_8 \leq 0$	✓	✓	✓	×
$\lambda_1 - \lambda_2 - \lambda_7 - \lambda_8 \leq 0$	✓	✓	✓	✓
$\lambda_1 - \lambda_3 - \lambda_6 - \lambda_8 \leq 0$	✓	✓	✓	✓
$\lambda_1 - \lambda_4 - \lambda_6 - \lambda_7 \leq 0$	✓	✓	✓	×
$\lambda_1 - \lambda_4 - \lambda_5 - \lambda_8 \leq 0$	✓	✓	✓	✓
$\lambda_3 - \lambda_4 - \lambda_7 - \lambda_8 \leq 0$	✓	✓	✓	×
$\lambda_2 - \lambda_4 - \lambda_6 - \lambda_8 \leq 0$	✓	✓	✓	×
$\lambda_2 + \lambda_3 + \lambda_5 - \lambda_8 \leq 2$	✓	✓	✓	×
$\lambda_1 + \lambda_3 + \lambda_6 - \lambda_8 \leq 2$	✓	✓	✓	✓
$\lambda_1 + \lambda_2 + \lambda_7 - \lambda_8 \leq 2$	✓	✓	✓	✓
$\lambda_1 + \lambda_2 + \lambda_3 - \lambda_4 \leq 2$	✓	✓	✓	×
$\lambda_1 + \lambda_4 + \lambda_5 - \lambda_8 \leq 2$	✓	✓	✓	✓
$\lambda_1 + \lambda_2 + \lambda_5 - \lambda_6 \leq 2$	✓	✓	✓	×
$\lambda_1 + \lambda_3 + \lambda_5 - \lambda_7 \leq 2$	✓	✓	✓	×

Table S2: Number of iterations required to achieve convergence using the pure and ensemble ADAPT-VQA methods. The calculations were performed on the $(4e, 3o)$ and $(4e, 4o)$ model systems for their 1-RDMs.

Algorithm	Iterations			
	$(4e, 3o)$		$(4e, 4o)$	
	$w = 0.0$	$w = 0.5$	$w = 0.0$	$w = 0.5$
pure	1	1	1	5
ensemble	1	1	1	1

Table S3: Number of iterations required to achieve convergence using the pure and ensemble ADAPT-VQA methods. The calculations were performed on the $(4e, 3o)$ and $(4e, 4o)$ model systems for their 2-RDMs.

Algorithm	Iterations			
	$(4e, 3o)$		$(4e, 4o)$	
	$w = 0.0$	$w = 0.5$	$w = 0.0$	$w = 0.5$
pure	1	1	1	18
ensemble	1	5	1	2

Table S4: Number of iterations required to achieve convergence using the pure and ensemble ADAPT-VQA methods. The calculations were performed on the $(4e, 4o)$ model system for its 1- and 2-RDMs with $w = 0.5$. The targets are the (ensemble) reduced density matrices corresponding to the states constructed from multiple substitutions. (See Table 4 in the main manuscript)

Substitution	Iterations			
	1-RDM		2-RDM	
	Algorithm			
	pure	ensemble	pure	ensemble
1	6	1	19	2
2	1	1	1	5
3	4	5	1	9

Table S5: Number of iterations required to achieve convergence using the ensemble ADAPT-VQA method for the 1- and 2-RDMs in the $(4e, 4o)$ model system. The targets are constructed by adding random noise of strength ε to the reduced density matrices (see main text for details).

ε	Iterations	
	1-RDM	2-RDM
0.0	1	1
10^{-2}	37	23
10^{-1}	48	34

Table S6: Number of iterations required to achieve convergence using the ensemble ADAPT-VQA method for the equilibrium and stretched H_2 and linear H_3 molecules. The targets are constructed by adding random noise of strength ε to the RDMs corresponding to canonical ensemble thermal states (see main text for details).

	Iterations			
	H_2		H_3	
	1-RDM	2-RDM	1-RDM	2-RDM
ε	R_{HH}=0.75 Å			
0.0	3	82	22	102
10^{-2}	11	90	27	72
10^{-1}	11	104	20	86
	R_{HH}=1.5 Å			
0.0	3	287	17	124
10^{-2}	8	293	25	152
10^{-1}	15	152	31	220
